# The response of different kinds of rapeseed cultivars to foliar application of nitrogen, sulphur and boron

**DOI:** 10.1038/s41598-021-00639-2

**Published:** 2021-10-26

**Authors:** Anna Sikorska, Marek Gugała, Krystyna Zarzecka

**Affiliations:** 1 Department of Agriculture, Vocational State School of Ignacy Mościcki in Ciechanów, ul. Narutowicza 9, 06-400 Ciechanów, Poland; 2 Faculty of Agrobioengineering and Animal Husbandry, University of Natural Sciences and Humanities in Siedlce, ul. Prusa 14, 08-110 Siedlce, Poland

**Keywords:** Plant sciences, Environmental impact

## Abstract

The study was based on in 2016–2017, 2017–2018, 2018–2019 field experiment conducted at the University of Natural Sciences and Humanities in Siedlce (Zawady Agricultural Experimental Station), eastern Poland. The studied factors were: I. winter rape cultivars: Monolit—open pollinated cultivar; PT248-F1 hybrid cultivars with traditional growth type; PX115-F1 hybrid cultivars with a semi-dwarf growth type and types of foliar nutrition: (1) control variant without foliar nutrition and amino acids; (2) amino acid; (3) foliar fertilizer sulphur and boron; (4) foliar fertilizer sulphur with foliar fertilizer boron and amino acid. The aim of the study was to determine the effect of foliar application of sulphur, boron, amino acids on the use and feed value of seeds of three winter rape morphotypes. The highest content of crude fat in seeds of the studied morphotypes was found after foliar fertilization with sulphur and boron and amino acids, while the lowest under the influence of amino acids. The highest concentration of total protein was obtained after the application of amino acids, and regardless of the morphotype studied on this object, the same value of this trait was demonstrated. In restored morphotypes, the use of additional foliar fertilization S and B in combination with amino acids did not significantly increase this characteristic compared to the amino acid variant. As a result of the application of amino acids and foliar feeding of S and B, and amino acids, the largest increase in crude fibre content in rapeseeds was obtained, while the application of S and B did not significantly increase this characteristic compared to the control variant. The best feed and use value of seeds were noted in restored morphotypes, with the semi-dwarf variety having the highest fat and crude fibre content. Climatic conditions in the years of research significantly determined the value of seeds. The highest values of the examined traits were obtained in the last year of the study, and the lowest in the growing season 2016–2017.

## Introduction

Winter rape (*Brassica napus*) is one of the most important oil-protein plants in the world^1–3^. The quality of rapeseed is determined by the content of mainly crude fat and total protein, as well as the concentration of harmful sulphur compounds called glucosinolates. Rapeseed is the main raw material for the production of edible oil and high-protein mean used in animal nutrition^4,5^.

The chemical composition of seeds depends mainly on the genetic factor, but it can also be shaped by environmental and agrotechnical conditions.

According to Szewczuk^6^, it is particularly beneficial to provide the plant with micronutrients in the foliar form. This form allows the supply of nutrients under difficult soil uptake and in the phases of the greatest demand for them. Winter rapeseed shows a high demand for sulphur and boron^7,8^. Kotecki et al.^9^ emphasize that sulphur is responsible for the synthesis of chlorophyll and amino acids, activates enzymes important in the metabolism of energy and fatty acids, increases the resistance of plants to diseases and pests, has a positive effect on the effectiveness of other nutrients, mainly nitrogen, and also limits the lodging of plants, while the boron determines the proper generative development in plants, the development of flowers, seeds and pods, and prevents the stems from cracking during rapid growth. Zao et al.^10^ showed that as a result of insufficient nutrition of plants with sulphur, the synthesis of sulphur amino acids is limited, which inhibits the process of protein formation and promotes the accumulation of non-protein forms of nitrogen. Amino acids are used as biostimulants, which, in addition to osmolytic activity, show a triple effect in reducing stress in plants, i.e. they play the role of metal chelator, antioxidant defense molecules and signal molecules^11^.

Rape seeds, despite their high energy and protein value, are not widely used in animal nutrition for economic reasons, and they are a raw material for the fat industry, and the by-products are used for fodder purposes. Hence, a higher protein and fat content is desired and a higher crude fibre content on the other hand now recognized as the major factor responsible for the poor digestibility of the rapeseed protein.

The application of foliar fertilizers, according to many authors, has a positive effect on the content of crude fat^7,12,13^ and total protein^4,5,14^.

The article adopts the research hypothesis that amino acids in combination with foliar feeding may have a positive effect on the quality of seeds of three winter rapeseed varieties. The aim of the research carried out in 2016–2019 was to determine the effect of foliar application of sulphur, boron, amino acids on the utility value (crude fat) and feed (total protein, crude fibre) of seeds of three winter rapeseed morphotypes (Monolit, PX115 and PT248).

## Materials and methods

### Arrangement of the experiment and research location

The study was based on a 3-year (2016–2019) field experiment conducted at the Zawady Agricultural Experimental Station (52°03′N and 22°33′E) belonging to the University of Natural Sciences and Humanities in Siedlce, eastern Poland. The field experiment was established in a split-plot design with three replicates. The area of one plot for harvest was 21 m^2^ ^15^.

### Factors of the experiment

The studied factors were:

#### I. Three winter rape cultivars

- Monolit (open pollinated cultivar);
- PT248 (F1 hybrid cultivars with traditional growth type);
- PX115 (F1 hybrid cultivars with a semi-dwarf growth type).

#### II. Four types of foliar nutrition

1. Control variant without foliar nutrition and amino acids;
2. Amino acid Aminoplant (amino acids, total nitrogen (N)—at least 8.5%): I date—in autumn in the phase of 4–6 leaves (BBCH 14–16), II date—in spring after the vegetation starts (BBCH 28–30), III date—in the phase of flower bud development (budding)—beginning of flowering (BBCH 50–61), at doses of 1.0 dm^3^ ha^−1^;
3. Foliar fertilizer Siarkomag (5% MgO, total SO_3_—85%, water-soluble SO_3_—10%) + foliar fertilizer Bormax (B—11%): I date—in autumn in the phase of 4–6 leaves (BBCH 14–16), II date—in spring after the vegetation starts (BBCH 28–30), III date—in the phase of flower bud development (budding)—beginning of flowering (BBCH 50–61), at doses of 2.0 dm^3^ ha^−1^ + 0.5 dm^3^ ha^−1^;
4. Foliar fertilizer Siarkomag + foliar fertilizer Bormax + amino acid Aminoplant: I date—in autumn in the phase of 4–6 leaves (BBCH 14–16), II date—in spring after the vegetation starts (BBCH 28–30), III date—in the flower bud development (budding)—beginning of flowering (BBCH 50–61), at doses of 2.0 dm^3^ ha^−1^ + 0.5 dm^3^ ha^−1^ + 1.0 dm^3^ ha^−1^.

Foliar fertilizers were applied with a plot sprayer with flat string nozzles. There were 300 dm^−3^ of working fluid per hectare; 150-cm wide paths were used between the plots^15^.

### Soil conditions

In the first year of research, spring wheat was the forecrop for winter rape, in the second and last year of research winter triticale. The experiment was carried out on soil classified according to the World Reference Base for Soil Resources^16^ to the Haplic Luvisol group, sanded, belonging to the very good rye soil complex, of the IVa class. In the years of the experiment, the pH (in 1 N KCl) of the soil was slightly acidic and ranged from 5.68 to 5.75. The soil was characterized by a low content of available forms of phosphorus (ranging from 75 to 81 mg kg^−1^) and average bioavailability in potassium (ranging from 199 to 202 mg kg^−1^), magnesium (ranging from 59 to 63 mg kg^−1^), boron (ranging from 0.48 to 0.52 mg kg^−1^), and sulphur (ranging from 5.90 to 6.03 mg SO_4_^2−^ kg^−1^)^15^.

### Fertilization

After harvesting the forecrop, a set of post-harvest cultivations was made with a stubble cultivator and a string roller, and then two weeks after the first treatment, the ploughing was carried out to a depth of 20 cm along with the ring roller. In order to prepare the soil for sowing and mixing fertilizers, a combined tilling set was used. Before sowing, phosphorus and potassium fertilization was applied at a rate of 40 kg P ha^−1^ and 110 kg K ha^−1^, and the first rate of nitrogen at 40 kg N ha^−1^. Fertilization under oilseed rape was applied in the form of Lubofos at a rate of 600 kg ha^−1^, i.e., 21 kg N ha^−1^, 26.4 kg P ha^−1^, 92.1 kg K ha^−1^, 34.8 kg S ha^−1^, and 1.2 kg B ha^−1^. Fertilization rates were supplemented with 55.9 kg ha^−1^ ammonium nitrate (19 kg N ha^−1^), 29.6 kg ha^−1^ triple superphosphate (13.6 kg P ha^−1^), and 29 kg ha^−1^ potassium salt (17.9 kg K ha^−1^). The second nitrogen rate of 100 kg ha^−1^ was applied in the spring before the start of growth (BBCH 28–30), using ammonium nitrate at a rate of 255.5 kg ha^−1^ (86.9 kg N ha^−1^) and ammonium sulphate at a rate of 62.5 kg ha^−1^ (13.1 kg N ha^−1^ + 15 kg S ha^−1^). The third nitrogen rate of 60 kg ha^−1^ was applied at the beginning of budding (BBCH 50) using ammonium nitrate at a rate of 176.5 kg ha^−1^ (60 kg N ha^−1^)^15^.

### Sowing

Sowing was carried out annually with a row spacing of 22.5 cm, assuming the sowing of 60 pcs m^-2^. Sowing was carried out at the optimal date recommended for this region (in 2016—August 12, 2017—August 14, and in 2018—August 13)^15^. The certified seed of the tested cultivars, which was used in the experiments, was obtained from Pioneer.

The tested cultivars and the biostimulants used in the experiment are listed in the COBORU National Register and the European Register. The experimental studies were performed in accordance with the relevant national and international guidelines and regulations.

### Ways of mechanical and chemical care

Chemical protection against weeds, diseases and pests was used in accordance with the recommendations of good agricultural practice. To control weeds, the Command 480 EC (0.25 dm^3^ ha^−1^) and Fusilade Forte 150 EG (2.0 dm^3^ ha^−1^, 13–14 BBCH) preparations were used. For pest control, Proteus 110 OD (0.6 dm^3^ ha^−1^, 30 BBCH, 50–58 BBCH, 60–69 BBCH) was used three times. Fungicide treatments were carried out using Horizon 250 EW (0.75 dm^3^ ha^−1^, 14–18 BBCH), Propulse 250 SE (1.0 dm^3^ ha^−1^, 61 BBCH) and Mondatak 450 EC w (1.0 dm^3^ ha^−1^, 65 BBCH)^15^.

### Seed collection

Rapeseed was harvested in two stages in the first and second decades of July (BBCH 89).

### Chemical analysis of seeds

Four seed samples for each variety were taken for chemical analysis. Samples of winter rape seeds were subjected to a chemical analysis for the content of:

- Crude fat (g kg^–1^ of dray matter—d.m.)—with the Soxhlet method, which extracted the fat with petroleum ether in a Soxhlet apparatus and determines its quantity by weight, total protein (g kg^–1^ of d.m.) PN-76/R-64753
- Total protein (g kg^–1^ of d.m.)—with the Kjeldahl method where protein nitrogen was converted to ammonium sulphate with concentrated sulphuric acid in the presence of a catalyst, the solution was alkalised, distilled and titrated with hydrochloric acid-ammonia bound with boric acid, the conversion factor Nx6.25 was used, crude fibre (g kg^–1^ of d.m.) PN-EN ISO 5983-2
- Crude fibres (g kg^–1^ of d.m.)—with the Wenden method consisting of the quantitative determination of organic substances insoluble during cooking in an acid solution.
- Chemical analyses of seeds were performed in the chemical-technological laboratory of the Experimental Variety Assessment Station in Słupia Wielka.

### Statistical analysis

The study results were statistically analysed by means of the analysis of variance. The significance of sources of variation was tested with the Fisher–Snedecor F test, and the significance of differences at the significance level α = 0.05 between the compared means was assessed using multiple Tukey intervals. Statistical calculations were made on the basis of our own algorithm written in Excel in accordance with the above mathematical model:

1 $${\mathrm{y}}_{\mathrm{ijl}}=\mathrm{m}+{\mathrm{a}}_{\mathrm{i}}+{\mathrm{g}}_{\mathrm{j}}+{\mathrm{e}}_{\mathrm{ij}}^{(1)}+{\mathrm{b}}_{\mathrm{l}}+{\mathrm{ab}}_{\mathrm{il}}+{\mathrm{e}}_{\mathrm{ijl}}^{(2)}$$

y_ijl_—value of the examined feature, m—population average, a_i_—the effect of the i-th level of factor A (cultivar), g_j_—the effect of the j-th repetition, ${e}_{ij}^{(1)}$—error 1 due to interaction of factor A and repetitions, b_l_—the effect of the l-th level of factor B (types of foliar feeding), ab_il_—the effect of interaction of factor A and B, ${e}_{ijl}^{(2)}$—random error.

## Results and discussion

### Total protein content depending on experimental factors

Based on our own research, it was found that foliar fertilization significantly increased the total protein content in rapeseed compared to the control object (Table 1). The largest increase in protein content by an average of 7.38 g kg^−1^ dray matter (d.m.) was obtained after the application of a biostimulant containing amino acids (object 2). Kozak et al.^17^, Matysiak et al.^18,19^ and Gugała et al.^20^ showed no significant effect of biostimulants on the value of this trait. In own research, the smallest increase on average from 2.52 g kg^−1^ d.m. was obtained after the application of foliar fertilizers containing sulphur and boron (object 3). Nawab et al.^21^ after foliar application of ammonium sulphate showed an increase in protein content on average from 0.3 to 0.5%. Similarly, Jankowski et al.^13^ after feeding plants with boron (300 g B ha^−1^) found a significant increase in protein content by an average of 8.8 g kg^−1^ d.m., while Bowszys^22^ on average by 18.0 g kg^−1^, while Jarecki et al.^23^ after application in autumn and twice in spring of the Insol 5 preparation—on average by 1.5% d.m. Different research results were obtained by Malhi et al.^5^ and Nadian et al.^24^, who did not show a significant effect of boron fertilization on the value of this trait. A similar tendency was noted by Jarecki and Bobrecka-Jamro^25^ after the application of foliar preparations: Basfoliar 36 Extra, Basfoliar 12 – 4 − 6 + S, Solubor DF, Adob Mn and the mixtures: Basfoliar 36 Extra with Solubor DF, Basfoliar 12 − 4 − 6 + S with Solubor DF, while Kwiatkowski^26^ after autumn spraying: 100% and 75% NPK and urea + nickel chelate + MgSO_4_ H_2_O; 100% and 75% NPK and urea + Plonvit R + MgSO_4_ H_2_O and Jankowski et al.^27^ after the autumn micro and macro-element feeding.

**Table 1:** Chemical composition of rapeseed depending on the types of foliar nutrition and varieties of foliar nutrition.

Types of foliar feeding		Cultivars			Mean
		Monolit	PT 248	PX 115	
**Total protein (g kg**^**−1**^** d.m.)**					
1	Control variant	370.99	374.82	375.14	373.65
2	Amino acid Aminoplant	380.17	380.93	382.00	381.03
3	Foliar fertilizer Siarkomag + foliar fertilizer Bormax	373.20	376.33	378.99	376.17
4	Foliar fertilizer Siarkomag + foliar fertilizer Bormax + amino acid Aminoplant	375.48	379.78	381.88	379.04
**Mean**		374.96	377.97	379.50	–
**LSD**_**0.05**_** for:**					
Cultivars					1.59
Types of foliar feeding					1.42
Interaction: cultivars × types of foliar feeding					2.46
**Crude fat (g kg**^**−1**^** d.m.)**					
1. Control variant		454.60	467.30	469.03	463.64
2. Amino acid Aminoplant		456.89	468.18	472.22	465.76
3. Foliar fertilizer Siarkomag + foliar fertilizer Bormax		461.58	470.68	474.63	468.96
4. Foliar fertilizer Siarkomag + foliar fertilizer Bormax + amino acid Aminoplant		464.98	473.58	477.41	471.99
**Mean**		459.51	469.93	473.33	**–**
**LSD**_**0.05**_** for**					
Cultivars					1.09
Types of foliar feeding					1.20
Interaction: cultivars × types of foliar feeding					2.00
**Crude fiber (g kg**^**−1**^** d.m.)**					
1. Control variant		81.64	81.26	82.91	81.94
2. Amino acid Aminoplant		82.90	82.42	84.68	83.33
3. Foliar fertilizer Siarkomag + foliar fertilizer Bormax		82.54	80.80	83.77	82.37
4. Foliar fertilizer Siarkomag + foliar fertilizer Bormax + amino acid Aminoplant		83.88	83.09	85.79	84.25
**Mean**		82.74	81.89	84.29	**–**
**LSD**_**0.05**_** for**					
Cultivars					0.57
Types of foliar feeding					0.99
Interaction: cultivars x types of foliar feeding					n.s

*d.m.* dray matter, *n.s.* not significant.

Seeds of hybrid cultivars (F1) with traditional growth type and semi-dwarf growth type (PT248 and PX115) had a higher protein content than the open pollinated cultivar (Table 1). In turn, Gugała et al.^19^ obtained the highest value of this feature in PX104 half-dwarf hybrid, while in the long-stemmed hybrid this value was, on average, smaller by 12.05 g kg^−1^ d.m. In subsequent studies of the authors^28^, the restored hybrid with the traditional growth type was characterized by the highest protein content, while the smallest by the semi-dwarf hybrid. Jarecki and Bobrecka-Jamro^25^ received a higher protein content in seeds of the Marita variety than in other varieties, and Ratajczak et al.^29^ did not show significant differences between hybrid morphotypes (F1) of traditional and semi-dwarf growth type, and the Califorium population variety.

Statistical calculations showed the interaction of cultivars with foliar feeding, which means that the cultivars studied showed a varied response to foliar fertilization (Table 1). In all morphotypes, the highest protein content was found after using the amino acid Aminoplant, while the differences between the morphotypes studied on this object were not statistically significant. In the case of hybrid morphotypes on plots with amino acid (2) and foliar fertilization with sulphur, boron and amino acids (4), the value of this trait was the same. It was shown that the seeds of the long-stemmed hybrid (PT248) were also characterized by the same protein content on the control object and after using sulphur and boron, while the population variety had the same protein content after using sulphur and boron (object 3) and sulphur, boron and amino acids (object 4).

### Crude fat content depending on experiment factors

The application of foliar fertilization resulted in a significant increase in crude fat content in rapeseeds on average from 2.12 to 8.35 g kg^−1^ d.m. compared to the control object (Table 1). The highest content of crude fat was demonstrated on object 4, after the application of foliar fertilizers containing sulphur, boron and amino acids, and the lowest after using a biostimulator containing amino acids (object 2). Malhi et al.^5^ came to similar conclusions, who showed, under the influence of foliar fertilizations containing B in the amount of 500 g ha^−1^, an increase in crude fat concentration in the seeds of *Brassica napus* and *Brassica rapa* on average 14.0 g kg^−1^ s.m. Jankowski et al.^13^ applying boron in a foliar manner at a dose of 150 g B ha^−1^ (BBCH 50) and 150 g B ha^−1^ (BBCH 55) noted an increase in the value of this trait by an average of 26.1 g kg^−1^ d.m, but after using a smaller dose (150 g B ha^−1^), the authors found no significant effect of the factor on the trait. In subsequent studies^27^ came to the conclusion that foliar application of macro and microelements made in autumn increased the content of raw fat by an average of 1.3 g kg^−1^ d.m. (one application) and 7.4 g kg^−1^ d.m. (two applications). A positive effect of foliar fertilization on the value of this trait was also demonstrated by Kwiatkowski^26^, who recorded an average increase from 16.0 to 29.0 g kg^−1^ d.m. In turn, Bowszys^22^ showed a reduction in the value in use of seeds after foliar feeding with boron in doses of 400, 600, 800, 1200 g B ha^−1^. Similar results were obtained in north-eastern Poland^30^ as a result of intensive foliar fertilization. Szczepanek et al.^31^ found little effect of the preparation: Humistar, Drakar and Humistar and Drakar (T3) on the value of this trait. The authors showed the beneficial effect of Drakar foliar fertilizer on the increase of oil content in seeds of the examined rapeseed varieties only in one growing season. While Sienkiewicz-Cholewa and Kieloch^8^ showed a beneficial effect of sulphur on the use value of seeds and no reaction to boron. Jarecki and Bobrecka-Jamro^23,25^ and Oleksy et al.^32^ after applying foliar fertilizers, received the same fat content as at the control object.

In own research, seeds of the PX115 semi-dwarf hybrid had the best utility value, and the smallest one—the open pollinated form Monolit. Similarly, Gugała et al.^28^ showed the smallest value of this trait in the open pollinated variety (Monolit), while in other studies of the authors^20^ the highest fat content was found in seeds of a restored hybrid with a semi-dwarf growth type, significantly lower by an average of 17.66 g kg^−1^ d.m. in the open pollinated form, while the smallest on average by 20.57 g kg^−1^ d.m. in a long-stemmed hybrid. Jarecki and Bobrecka-Jamro^25^ showed statistically insignificant differences in fat content between the Lirajet, Lisek and Marita varieties, while Oleksy et al.^32^ found a higher value of this trait in a hybrid Nelson.

The cultivars were distinguished by a varied response to foliar nutrition (Table 1). Seeds of the studied cultivars had the highest crude fat content after application of foliar sulphur, boron and amino acids. The long-stem cultivar PT 248, after applying the amino acid, had the same value of this trait as on the control object.

### Crude fibre content depending on experimental factors

On the basis of own research, it was shown that as a result of the application of foliar fertilizers containing S and B, the same raw fibre content was obtained as at the control object. After the application of S and B in combination with amino acids, the highest increase was shown on average by 2.31 g kg^−1^ d.m. of the value of this feature (Table 1), with the differences between this object and the object on which only amino acids were used, were statistically insignificant. Jankowski et al.^13^ showed a significant increase in the content of crude fibre after the application of foliar fertilizers containing boron (300 g B ha^−1^), while in later studies^27^ after the autumn foliar feeding, the authors showed a low impact of this factor. In turn, Jarecki and Bobrecka-Jamro^25^ found that foliar nutrition of rapeseed did not significantly affect the content of crude fibre in rape seeds, while in subsequent studies the authors^23^ under the influence of foliar fertilizers applied in autumn and spring, in autumn and twice in spring, twice in spring found a reduction in fibre content of seeds of 0.6%, on average.

Of the compared varieties, the most crude fibre was found in a semi-dwarf hybrid, while the smallest in the hybrid with the traditional type of growth. This is in line with the results of the research by Gugała et al.^20^, while Jarecki and Bobrecka-Jamro^25^ showed statistically insignificant differences in crude fibre content between the Lirajet, Lisek and Marita varieties.

### Use and feed value of seeds depending on climatic conditions

**Figure 1:**
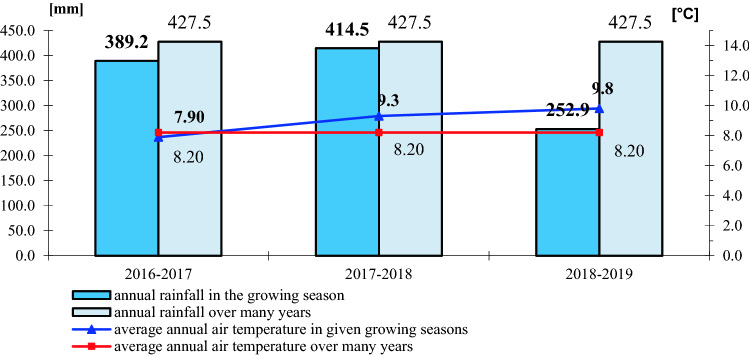
Monthly precipitation sums and average air temperature in given growing seasons RSD Zawady of many years (1996–2010).

**Figure 2:**
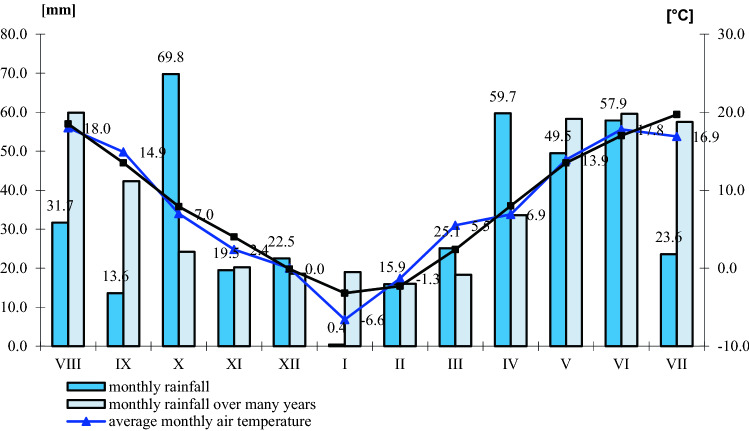
Monthly rainfall totals and average air temperature during the growing season 2016–2017 in RSD Zawady of many years of many years (1996–2010).

**Figure 3:**
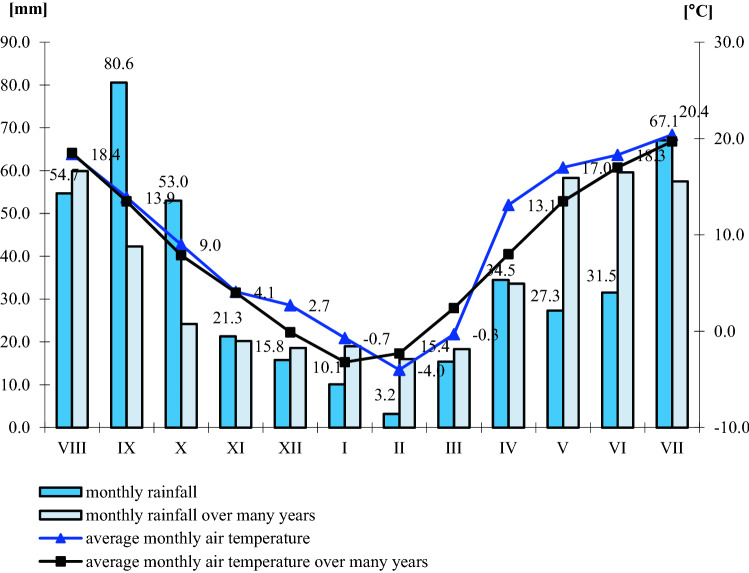
Monthly rainfall totals and average air temperature during the growing season 2017–2018 in RSD Zawady of many years (1996–2010).

**Figure 4:**
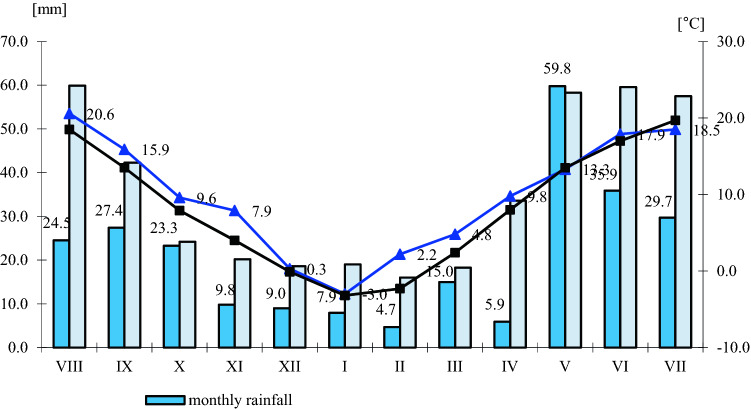
Monthly rainfall totals and average air temperature during the growing season 2018–2019 in RSD Zawady of many years (1996–2010).

In the years of the experiment, climatic conditions influenced the chemical composition of winter rapeseed (Figs. 1, 2, 3, and 4). The highest annual rainfall (on average 414.5 mm) was recorded in the 2017–2018 growing season, the average annual air temperature was 9.3 °C (Fig. 1). In terms of the calculated Sielianinov coefficient, it was the optimal season (K = 1.44). The last year of research was the warmest and driest (K = 0.75) (Table 2). The annual rainfall was 13.0 mm lower than the multi-annual average, while the average annual air temperature was 1.1 °C higher than the 1996–2010 average.

**Table 2:** Characteristics of weather conditions in the years 2016–2019 (Zawady Meteorological Station, Poland).

Years	Months								Mean
	VIII	IX	X	III	IV	V	VI	VII	
	Sielianinovs hydrothermic coefficients*								
2016–2017	0.61	0.28	3.02	1.79	3.19	1.52	1.06	0.47	1.49
2017–2018	1.00	1.92	2.36	2.97	0.99	0.59	0.61	1.12	1.44
2018–2019	0.40	0.71	0.94	1.16	0.20	1.37	0.63	0.56	0.75

*Index value^33^: extremely dry k ≤ 0.4. very dry 0.4 < k ≤ 0.7. dry 0.7 < k ≤ 1.0. rather dry 1.0 < k ≤ 1.3. optimal 1.3 < k ≤ 1.6. rather humid 1.6 < k ≤ 2.0. humid 2.0 < k ≤ 2.5. very humid 2.5 < k ≤ 3.0. extremely humid k > 3.0.

Based on the experiment, it was shown that the seeds of the studied morphotypes were distinguished by the highest protein content in the driest and warmest years of studies, with the highest value of this trait shown in the long-stemmed hybrid PT248. In the growing seasons of 2016–2017 and 2017–2018, differences between the open pollinated morphotype and the long-stemmed hybrid were statistically insignificant. In the second and third year of research, the same value of the trait was also shown between the open pollinated form and the semi-dwarf hybrid. The least protein in the seeds of all studied variables was demonstrated in the first year of the study (Table 3).

**Table 3:** Chemical composition of rapeseed depending on the cultivars studied and climatic conditions in the years of the study.

Years	Cultivars			Mean
	Monolit	PT 248	PX 115	
**Total protein (g kg**^**−1**^** d.m.)**				
2016–2017	351.75	353.26	367.00	357.34
2017–2018	371.68	373.12	370.15	371.65
2018–2019	401.45	407.53	401.36	403.44
**Mean**	374.96	377.97	379.50	–
**LSD**_**0.05**_** for**				
Cultivars				1.59
Years				1.59
Interaction: cultivars × years				2.76
**Crude fat (g kg**^**−1**^** d.m.)**				
2016–2017	448.38	465.09	467.27	460.24
2017–2018	457.77	469.73	470.32	465.94
2018–2019	472.39	474.98	482.39	476.59
**Mean**	459.51	469.93	473.33	
**LSD**_**0.05**_** for**				
Cultivars				1.09
Years				1.09
Interaction: cultivars × years				1.89
**Crude fiber (g kg**^**−1**^** d.m.)**				
2016–2017	79.48	77.13	82.23	79.61
2017–2018	81.52	83.93	84.25	83.23
2018–2019	82.23	84.61	86.38	86.07
**Mean**	82.74	81.89	84.29	−
**LSD**_**0.05**_** for**				
Cultivars				0.57
Years				0.57
Interaction: cultivars × years				0.99

*d.m.* dray matter, *n.s.* not significant.

In all varieties tested, the highest crude fat content was found in the last year of studies, and the lowest in the growing season 2016–2017. In the second year of research, the same value of this trait was shown in the restored hybrids (Table 3).

The seeds of the studied cultivars collected in the last year of the study had the highest content of crude fibre, while in the growing season 2017–2018 and 2018–2019 the value of this trait was the same for the open pollinated variety. A similar tendency was demonstrated in the restored hybrid with the traditional growth type. In the second year of the study, statistically insignificant differences were found between restored hybrids with traditional and semi-dwarf growth types. The lowest crude fibre content in all studied varieties was demonstrated in 2016–2017 (Table 3).

In all years of research, the highest protein content was obtained after using amino acids (Table 4). In the first year of research after applying S and B feeding, the protein content was the same as for the control object. This year, the same value of this trait was demonstrated o objects where foliar fertilization (S and B) as well as sulphur and boron was used in combination with amino acids, and on objects with fertilizers containing S, B, amino acids and where only amino acids were used. In the growing season 2017–2018, statistically insignificant differences were found on the control object and with foliar fertilizers containing S and B, as well as on objects 3 (S, B) and 4 (S, B, amino acids).

**Table 4:** Chemical composition of rapeseed depending on climatic conditions in the years of the study and types of foliar nutrition.

Types of foliar feeding	Years			Mean
	2016–2017	2017–2018	2018–2019	
**Total protein (g kg**^**−1**^** d.m.)**				
1. Control variant	354.84	368.84	397.27	373.65
2. Amino acid Aminoplant	359.66	375.21	408.23	381.03
3. Foliar fertilizer Siarkomag + foliar fertilizer Bormax	356.43	371.00	401.09	376.17
4. Foliar fertilizer Siarkomag + foliar fertilizer Bormax + amino acid Aminoplant	358.41	371.53	407.19	379.04
**Mean**	357.34	371.65	403.44	−
**LSD**_**0.05**_** for**				
Years				1.59
Types of foliar feeding				1.42
Interaction: years × types of foliar feeding				2.46
**Crude fat (g kg**^**−1**^** d.m.)**				
1. Control variant	455.39	462.63	472.91	463.64
2. Amino acid Aminoplant	458.40	464.61	474.28	465.76
3. Foliar fertilizer Siarkomag + foliar fertilizer Bormax	462.08	466.94	477.87	468.96
4. Foliar fertilizer Siarkomag + foliar fertilizer Bormax + amino acid Aminoplant	465.11	469.56	481.30	471.99
**Mean**	460.24	465.94	476.59	
**LSD**_**0.05**_** for**				
Years				1.09
Types of foliar feeding				1.42
Interaction: years × types of foliar feeding				n.s
**Crude fiber (g kg**^**−1**^** d.m.)**				
1. Control variant	79.34	81.78	84.69	81.94
2. Amino acid Aminoplant	79.98	82.83	87.19	83.33
3. Foliar fertilizer Siarkomag + foliar fertilizer Bormax	78.62	83.26	85.23	82.37
4. Foliar fertilizer Siarkomag + foliar fertilizer Bormax + amino acid Aminoplant	80.51	85.07	87.18	84.25
**Mean**	79.61	83.23	86.07	–
**LSD**_**0.05**_** for:**				
Years				0.57
Types of foliar feeding				0.99
Interaction: years × types of foliar feeding				1.60

*d.m.* dray matter, *n.s.* not significant.

In all years of conducting the experiment, the content of crude fibre was the highest under the influence of foliar fertilizers containing sulphur and boron in combination with amino acids. In the first and second year of research on the control object (1), with the Aminoplant biostimulant (2) and with foliar fertilization with S and B (3), the value of this trait was the same. A similar relationship was observed in the second year of the study, while in the last one the crude fibre content was the same as for the control object after the application of foliar fertilizers containing sulphur and boron. In the growing season 2018–2019, statistically insignificant differences were found on the object with amino acids and where foliar fertilization with boron, sulphur and amino acids was used (Table 4).

## Conclusions

1. The highest content of crude fat in seeds of the studied morphotypes was found after foliar fertilization with sulphur and boron and amino acids, while the lowest under the influence of amino acids.
2. The highest concentration of total protein was obtained after the application of amino acids, and regardless of the morphotype studied on this object, the same value of this trait was demonstrated. In restored morphotypes, the use of additional foliar fertilization S and B in combination with amino acids did not significantly increase this characteristic compared to the amino acid variant. As a result of the application of amino acids and foliar feeding of S and B, and amino acids, the largest increase in crude fibre content in rapeseeds was obtained, while the application of S and B did not significantly increase this characteristic compared to the control variant.
3. The best feed and use value of seeds were noted in restored morphotypes, with the semi-dwarf variety having the highest fat and crude fibre content.
4. Climatic conditions in the years of research significantly determined the value of seeds. The highest values of the examined traits were obtained in the warmest and driest research year, and the lowest in the growing season, which was distinguished by a much higher rainfall compared to the multi-year average in the period from spring start of vegetation to technical maturity of seeds
